# Metabolism-Related Bioinformatics Analysis Reveals That HPRT1 Facilitates the Progression of Oral Squamous Cell Carcinoma In Vitro

**DOI:** 10.1155/2022/7453185

**Published:** 2022-05-09

**Authors:** Hengyu Ye, Zenan Zheng, Yuxing Song, Guangzhao Huang, Qingqing Wu, Yilong Ai, Xiaozhi Lv

**Affiliations:** ^1^Nanfang Hospital, Southern Medical University, Guangzhou, Guangdong, China; ^2^Foshan Stomatological Hospital, School of Stomatology and Medicine, Foshan University, China

## Abstract

**Objectives:**

Many studies have shown that dysregulation of metabolism contributes to oncogenesis. However, the exact roles of metabolism-related genes (MRGs) in oral squamous cell carcinoma (OSCC) remain unclear. Thus, we aimed to identify a prognostic signature related to MRGs in OSCC.

**Methods:**

The gene sequencing data of OSCC samples and the MRG set were downloaded from The Cancer Genome Atlas (TCGA) and the Molecular Signatures Database (MSigDB). The Wilcoxon rank-sum test was used to identify differentially expressed MRGs. Then, a prognostic signature was established by multivariate Cox regression analysis. Finally, prognosis-related MRGs were selected and further validated in OSCC tissues and cell lines.

**Results:**

A prognostic signature that included 8 MRGs was constructed. Multiple survival analysis revealed that only HPRT1 might be an independent biomarker and indicator of poor overall survival in OSCC patients. The expression of HPRT1 was then found to be upregulated in OSCC tissues and cell lines, and suppression of HPRT1 gene expression by siRNA inhibited the proliferation, migration, and invasion of OSCC cells in vitro.

**Conclusions:**

MRGs play an important role in the development of OSCC. Furthermore, HPRT1 might be an independent biomarker of OSCC and enhance OSCC proliferation, migration, and invasion in vitro; these results emphasize the potential utility of HPRT1 in OSCC therapy.

## 1. Introduction

Oral squamous cell carcinoma (OSCC) is the most common type of oral cancer and has a critical impact on the quality of life of patients around the world [1]. Cigarette smoking, alcohol consumption, and betel nut consumption are the most critical risk factors for OSCC [2]. Interestingly, a recent investigation showed that the human microbiome might also be a potential risk factor and could play an important role in early OSCC detection [3]. The oral cavity is an ideal place for microbiome growth due to its temperature (37°C), pH (6.5~7.5), and hard tooth surfaces. Among numerous microorganisms, Streptococcaceae species dominate most of oral habitats [4]. Recent research discovered that *Porphyromonas gingivalis* can cause oral cancer, for example, by reducing T cell proliferation [5]. Although few studies have focused on the relationship between the oral microbiome and OSCC, it is clear that the microbiome at the tumor site is more diverse than that of the healthy oral mucosa [3].

The basic options for treating OSCC are surgery, radiation therapy (RT), chemotherapy, or a combination of these therapeutic methods. The harm caused by RT to patients cannot be ignored. With the development of technology, some interventions are claimed to minimize harm to patients; however, there is not enough evidence that these interventions actually reduce the impact on patients [6]. Xerostomia, trismus, fibrosis and muscle atrophy, caries, and osteoradionecrosis often occur after the implementation of radiation therapy protocols. Therefore, in addition to improvements to treatment techniques, care for patients after treatment should also receive attention [7].

Lymph node metastasis and recurrence result in an unfavorable overall 5-year survival rate of OSCC, ranging from only 45 to 50% [8]. Therefore, follow-up programs for OSCC patient management are urgently needed. However, the best follow-up strategy for OSCC remains controversial. Experts have formulated follow-up plans for each primary tumor subgroup according to their daily clinical practice [9]. This approach will help to develop proper follow-up strategies for different patients. Considering the unfavorable 5-year survival rate and the severe impact on patients, it is necessary to better understand the potential mechanisms underlying the initiation and development of OSCC [10].

It is known that normal cell metabolism is dependent on normal signaling pathways and basic metabolites [11]. Many studies have revealed that tumorigenesis relies on cellular metabolism reprogramming as a direct or indirect result of oncogenic mutations [12]. In the 1920s, Warburg initially observed that cancer cells actively absorb glucose and turn pyruvate into lactate despite the availability of sufficient oxygen levels; this phenomenon is now known as aerobic glycolysis [13]. This process creates an environment conducive to tumor cell survival and proliferation and dramatically affects the tumor microenvironment. Currently, it is widely accepted that the metabolic mechanisms play crucial roles in the initiation and development of cancer. Studies have shown that metabolism-related genes are involved in lung cancer [14], gastric cancer [15], and liver cancer [16]. However, only a few investigations have focused extensively on the association of metabolic genes with OSCC.

In this study, we aimed to evaluate the differentiated profiles of MRG expression in OSCC and develop a Cox regression model to predict the overall survival of OSCC patients. Ultimately, the function of HPRT1, a selected differentially expressed MRG, was investigated in vitro. This study may provide insight into the molecular mechanism underlying OSCC and provide a novel potential therapeutic target for OSCC.

## 2. Materials and Methods

### 2.1. Data Collection and Preprocessing

The transcriptomic data of OSCC including oral cavity, floor of the mouth, palate, buccal mucosa, the anterior 2/3 of the tongue, and gingiva were obtained from TCGA (https://portal.gdc.cancer.gov/) database. The RNA-seq data were evaluated, including 312 tumor cases and 39 neighboring nontumor cases from TCGA database. The MRGs were found using gene set enrichment analysis (GSEA) on the metabolic pathway-related gene sets “c2.cp.kegg.v7.0.symbols.” MRGs can be studied further if included in the two datasets above.

### 2.2. Differentially Expressed MRGs

MRGs with differential expression were identified in OSCC and normal oral tissues using the R package “limma” and the Wilcoxon test method [17]. Results were considered significant at |log*FC*| >1 and adjusted *p* < 0.05. The heatmap and volcano plot were constructed by using the ggplots2 package in R software.

### 2.3. Gene Ontology and Kyoto Encyclopedia of Gene Enrichment Analysis

Gene Ontology (GO) and Kyoto Encyclopedia of Genes and Genomes (KEGG) pathway enrichment analysis were carried out with the R package clusterProfiler to investigate the biological function and possible pathways of these MRGs. The functional categories were provided with a false discovery rate (FDR) of less than 0.05.

### 2.4. Protein-Protein Interaction Network Construction

The STRING database was used to map the differentially expressed MRGs, resulting in an interactive network that displays gene connections. The protein-protein interaction network was then visualized using the Cytoscape software. The cytoHubba plugin was utilized to discover a hub gene in this complete network.

### 2.5. Prognostic Model Construction and Evaluation

All OSCC samples were randomly divided into a training set (*n* = 224) and a validation set (*n* = 88). The training set was used to build the prognosis model, while the validation set was used to test it. The primitive data for metabolism-related genes were converted and normalized in a log2(*x* + 1). Univariate Cox regression was used to select prognosis-related factors. We then used the R package to do Cox regression analysis paired with LASSO regression to create a risk model. The cross-validation method chose the penalty regularization parameter lambda (*λ*) with an *n*-fold equal to 10. In the meantime, lambda Cox regression analysis was used to select the variables. Finally, according to the risk score for each patient, eight metabolism-related genes were included in risk Cox regression and survival analysis, scatter diagram, and heatmap in R software [18]. Furthermore, univariate and multivariate Cox regression was used to see if the risk score was a prognostic factor on its own [19]. The validation set was also used to analyze the prognostic model to verify its value. Ultimately, the gene expression profile from GEO (Gene Expression Omnibus) was used to verify these biomarkers.

### 2.6. ROC Analysis

A receiver operating characteristic (ROC) curve analysis was performed to assess the sensitivity and specificity of the MRGs for OSCC diagnosis by using the R package. The area under the curve (AUC) value was calculated and used [20].

### 2.7. Gene Set Enrichment Analysis

GSEA version 2-2.2.3 (JAVA) [21] and MSigDB version v6.2 (Molecular Signatures Database) were downloaded from the Gene Set Enrichment Analysis website (http://software.broadinstitute.rg/gsea/index.jsp). Using the default weighted enrichment statistical method, the approach was made 1000 times for each analysis. We identified gene sets with a false discovery rate (FDR) < 0.25 and a family‐wise error rate < 0.05.

### 2.8. Patients and Sample Collection

20 pairs of oral squamous cell carcinoma specimens and normal adjacent tissues were collected at Nanfang Hospital, Southern Medical University (Guangzhou, China), and written informed consent was obtained from all patients.

### 2.9. Cell Culture

The human OSCC cell lines scc9, scc15, scc25, and cal27 and the normal oral epithelial cell line HOK were obtained from the Institute of Antibody Engineering, Southern Medical University (Guangzhou, China). HOK was cultivated in MEM (Gibco, Cat#C12571500BT-10), scc9 in Dulbecco's modified Eagle's medium F12 (DMEM/F12) (Gibco, Cat#C11330500BT), scc15 and scc25 in DMEM (Gibco, Cat#11995500TB), and cal27 in *α*-MEM (Gibco, Cat#C1257100BT-10).

### 2.10. RNA Extraction and RT-qPCR

Total RNA was isolated from cells and tissues according to the manufacturer's instructions for TRIzol (Takara, Cat# 9109). The manufacturer followed the Reverse Transcription Kit (Vazyme, Cat# R212-02) to reverse the total RNA to cDNA. The expression levels of each gene were standardized to GAPDH. Experiments were carried out in triplicate, with the results shown as mean values with standard deviations. The following are the primer sequences in detail:

GAPDH: forward primer (5′-3′): CGCTGAGTACGTCGTGGAGTC; reverse primer (5′-3′): GCTGATGATCTTGAGGCTGTTGTC

HPRT1: forward primer (5′-3′): CCTGGCGTCGTGATTAGTGAT; reverse primer (5′-3′): AGACGTTCAGTCCTGTCCATAA

### 2.11. Immunohistochemical Analysis

OSCC and normal tissue samples were fixed with 4% formaldehyde, dehydrated, embedded in paraffin, and ultimately sectioned into 4 *μ*m sections. Dewaxing and rehydration of tissue section in xylene and graded ethanol were performed. After that, slices were treated with 3% hydrogen peroxide for 10 minutes to inhibit endogenous peroxidase. Following that, 15 minutes in a pressure cooker with 0.01 M citrate buffer (pH 6.0) was used to accomplish antigen retrieval. The sections were treated overnight at 4°C with the primary antibody and 1 hour at room temperature with the secondary antibody. Finally, 3,3′-diaminobenzidine (DAB) was used to visualize the sections.

### 2.12. Western Blotting

RIPA lysis buffer was used to lyse cells. Proteins were separated by electrophoresis, transferred to membranes, and then sealed with 5% skim milk. HPRT1 (dilution 1 : 1000) and GADPH (dilution 1 : 1000) primary antibodies were incubated in 4°C for one night. Following that, goat anti-mouse and goat anti-rabbit secondary antibodies were incubated for 1 hour at room temperature.

### 2.13. Cell Transfection

siRNAs (TINGKE, 100 mM) for HPRT1 were transfected into cells using Lipofectamine 3000 (Invitrogen, Cat# L3000-015). Transfection was performed in 6-well plates with 2500 ng siRNA. After 2-4 days, RNAs were collected. Additionally, the siRNA sequence was as follows:

SI-HPRT1-1: sense 5′-3′: GCCCUUGACUAUAAUGAAUTT; antisense 5′-3′: AUUCAUUAUAGUCAAGGGCTT

SI-HPRT1-2: sense 5′-3′: CCCACGAAGUGUUGGAUAUTT; antisense 5′-3′: AUAUCCAACACUUCGUGGGTT

SI-HPRT1-3: sense 5′-3′: CCUGCUGGAUUACAUCAAATT; antisense 5′-3′: UUUGAUGUAAUCCAGCAGGTT

### 2.14. Colony Formation Assay

Cells were inoculated in 12-well plates at a density of 2000 cells/well and incubated under 37°C and 5% CO_2_ conditions for one week. One week later, the cells were washed with phosphate-buffered saline (PBS), fixed in 1 mL/well 4% paraformaldehyde (Leagene Biotechnology, Beijing, China) for 20 min, and stained with 1% crystal violet staining solution (Solarbio, Beijing, China) for 10 min at room temperature. Finally, the crystal violet staining solution was slowly washed off with running water and dried in the air.

### 2.15. Transwell Assay

The Matrigel 1 : 8 dilution was coated at the bottom of the chamber and put at 37°C for 2 hours. 5∗10^4^ transfected cells were suspended in 200 *μ*L serum-free medium per well and seeded in the upper chambers. 600 *μ*L DMEM or DMEM/F12 with 10% FBS was placed to the bottom wells. In addition, transfected cells were cultured at 37°C with 5% CO_2_ for 24-72 hours. Subsequently, the cells in the upper side were removed with a cotton swab, and the migration and invasion cells were fixed with 4% formaldehyde and then stained with crystal violet. Finally, the number of migration and invasion cells was counted by using an inverted microscope.

### 2.16. Wound Healing Assay

The transfected cells were cultured in 6-well plates at a density of 5∗10^5^ cells/well. When cells grew until reaching a confluence of 90%, a linear wound was generated across the cell monolayer by using a sterile 200 *μ*L pipette tip. Furthermore, the cells were washed with PBS 3 times to remove floating cells or debris and then cultured in a serum-free medium with 5% CO_2_ at 37°C for additional 12-24 hours. Images were taken at 0 and 24 hours under an inverted phase-contrast microscope.

### 2.17. Statistical Analysis

SPSS23.0 software (IBM) was used to conduct all statistical analyses. Statistical significance was determined by Student's *t*-test. The log-rank test was used to compare the Kaplan-Meier survival curves. Data were considered statistically significant if the *p* value was less than 0.05.

## 3. Results

### 3.1. Identification of Differentially Expressed MRGs and Functional Analysis

**Figure 1:**
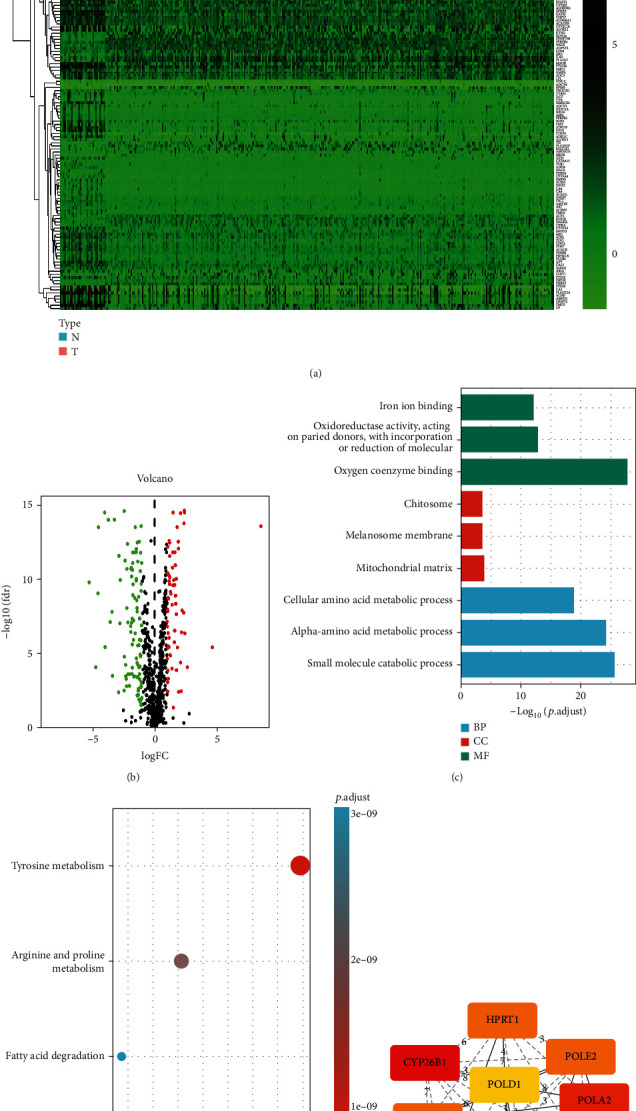
Identification of MRGs. (a) Heatmap of MRGs between 312 tumor tissues and 39 normal oral tissues in TCGA database. (b) Volcano plot of MRGs in TCGA database. The red dots in the plot represent significantly upregulated genes, and the green dots represent significantly downregulated genes. (c) Enriched GO terms. (d) Enriched KEGG pathways. (e) The top 10 MRGs with the highest degree of connectivity were determined to be hub genes.

A total of 170 differentially expressed MRGs were identified using the cutoff criteria (adjusted *p* value < 0.05 and |log *FC*| > 1.0), and these differentially expressed MRGs included 104 downregulated and 66 upregulated MRGs (Figures 1(a) and 1(b)). Although we know that these genes are involved in metabolism, GO and KEGG analyses were essential to explore the specific pathways and biological functions of these MRGs. GO analysis revealed that the differentially expressed MRGs were predominantly enriched in biological processes, such as iron ion binding, oxidoreductase activity, and oxygen coenzyme binding (Figure 1(c)). The enriched cellular component terms revealed that the differentially expressed MRGs were mostly related to the chitosome, melanosome membrane, and mitochondrial matrix (Figure 1(c)). For molecular function, the selected MRGs were mainly enriched in cellular amino acid metabolic processes, alpha-amino acid metabolic processes, and small molecule catabolic processes (Figure 1(c)). In addition, KEGG pathway analysis showed that differentially expressed MRGs were associated with tyrosine metabolism, arginine and proline metabolism, and fatty acid degradation pathways (Figure 1(d)). Then, we built a protein-protein interaction network to explore the connections of these MRGs. The confidence score was greater than 0.7. Hub genes were selected through cytoHubba's DMNC algorithm, and the top 10 genes are shown (Figure 1(e)). The most significant hub gene was CYP26B1, with a score of 0.618139, followed by POLA2 (score = 0.583436) and HPRT1 (score = 0.57059). Genes with high scores were also highly correlated with other genes and might play key roles in metabolism-related pathways; these findings will be the focus of future research.

### 3.2. Establishment and Validation of a Metabolism-Related Prognostic Signature for OSCC

**Figure 2:**
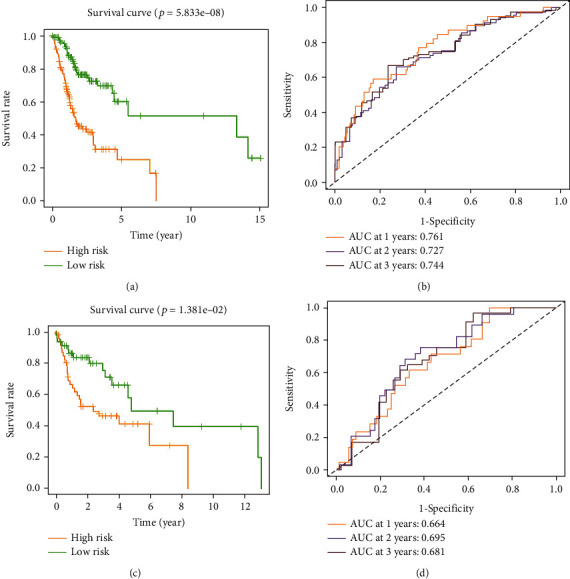
Construction and validation of a metabolism-based prognostic signature. (a) Survival curves of the low-risk and high-risk groups in the training set. (b) Time-independent receiver operating characteristic (ROC) analysis of risk scores for the prediction of overall survival (OS) in the training set. (c) Survival curves of the low-risk and high-risk groups in the validation set. (d) Time-independent receiver operating characteristic (ROC) analysis of risk scores for the prediction of OS in the validation set.

A univariate Cox regression analysis was initially performed on 170 differentially expressed MRGs in the training group, and this analysis revealed that 9 MRGs were significantly associated with OS (Supplement Figure 1A). Then, Lasso Cox regression analysis was used to determine variables (Supplement Figures 1B and 1C). Finally, eight variables were selected (Supplement Figure 1D), including ASPA, HPRT1, CA9, ADH7, AGPAT4, CHDH, ADA, and PCK1. Risk score = (2.164481∗ASPA) + (0.019928∗HPRT1) + (0.008596∗CA9) + (0.010405∗ADH7) + (0.257026∗AGPAT4) + (−0.88363∗CHDH) + (0.018996∗ADA) + (0.980108∗PCK1). The risk score for each patient was then calculated using this prognostic model. A total of 224 OSCC patients were divided into a high-risk group (*n* = 112) and a low-risk group (*n* = 112) based on the median risk score. The Kaplan-Meier curve and log-rank test revealed that patients in the high-risk group had significantly shorter life expectancy than those in the low-risk group (Figure 2(a)). The areas under the curve values of the prediction of the 1-, 2-, and 3-year OS rates by the signature were 0.761, 0.727, and 0.744, respectively, indicating that this prognostic model had good sensitivity and specificity (Figure 2(b)). The validation set was used to analyze the prognostic model to verify its value. The survival analysis based on the validation set revealed that the OS of the high-risk group was more unfavorable than that of the low-risk group (Figure 2(c)). The AUCs of the validation set for 1-, 2-, and 3-year OS rates were 0.664, 0.695, and 0.681, respectively (Figure 2(d)), suggesting that in both the training and validation sets, the prognostic model performed well. In addition, the GSE37991 dataset was used to validate the mRNA expression levels of the eight genes. The results showed that HPRT1, CA9, AGPAT4, CHDH, and ADA expression in OSCC tissues was higher than that in nearby normal oral tissues, while ADH7, ASPA, and PCK1 expression was lower in OSCC tissues than in adjacent tissues (Supplement Figure 2).

### 3.3. Clinical Value of the Prognostic Signature

**Figure 3:**
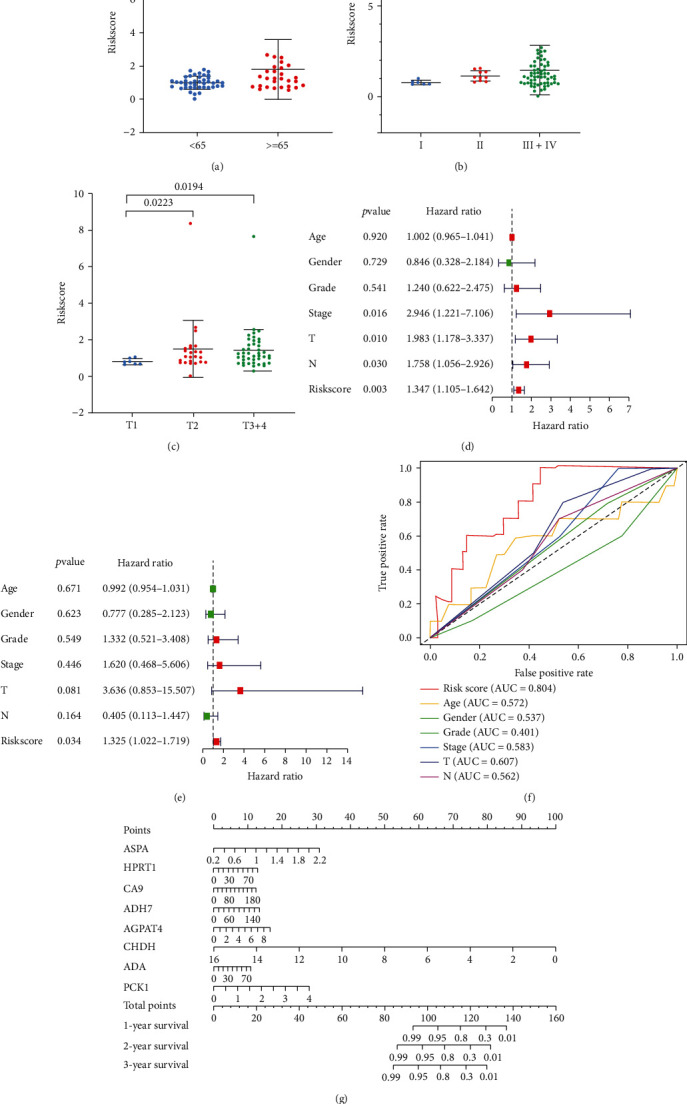
Clinical value of the prognostic signature. (a) The relationship between age and risk score. (b) The relationship between the stage and risk score. (c) The relationship between T classification and risk score. Univariate (d) and multivariate (e) independent prognostic analyses of independent risk factors for OS in patients with OSCC. (f) Receiver operating characteristic (ROC) analysis of the risk score and other clinical features. (g) Construction of a nomogram for the signature at 1, 2, and 3 years.

Statistical analysis was performed to investigate whether this prognostic model was associated with other clinical characteristics of OSCC patients. The results showed that the risk score was higher in older patients (Figure 3(a)) and in patients with higher clinical stage and T classification (Figures 3(b) and 3(c)). Univariate and multivariate Cox regression analyses were used to assess the independent prediction ability of the metabolism-related prognostic signature (Figures 3(d) and 3(e)). The results revealed that only the metabolism-related prognostic signature could be used as an independent prognostic factor. The AUC values of the risk score were higher than those of other clinicopathologic characteristics (Figure 3(f)). The eight genes in this signature were used to create a nomogram for predicting 1-, 2-, and 3-year OS in OSCC patients (Figure 3(g)).

### 3.4. Identification of Biomarkers Related to MRGs for the Diagnosis of OSCC

**Figure 4:**
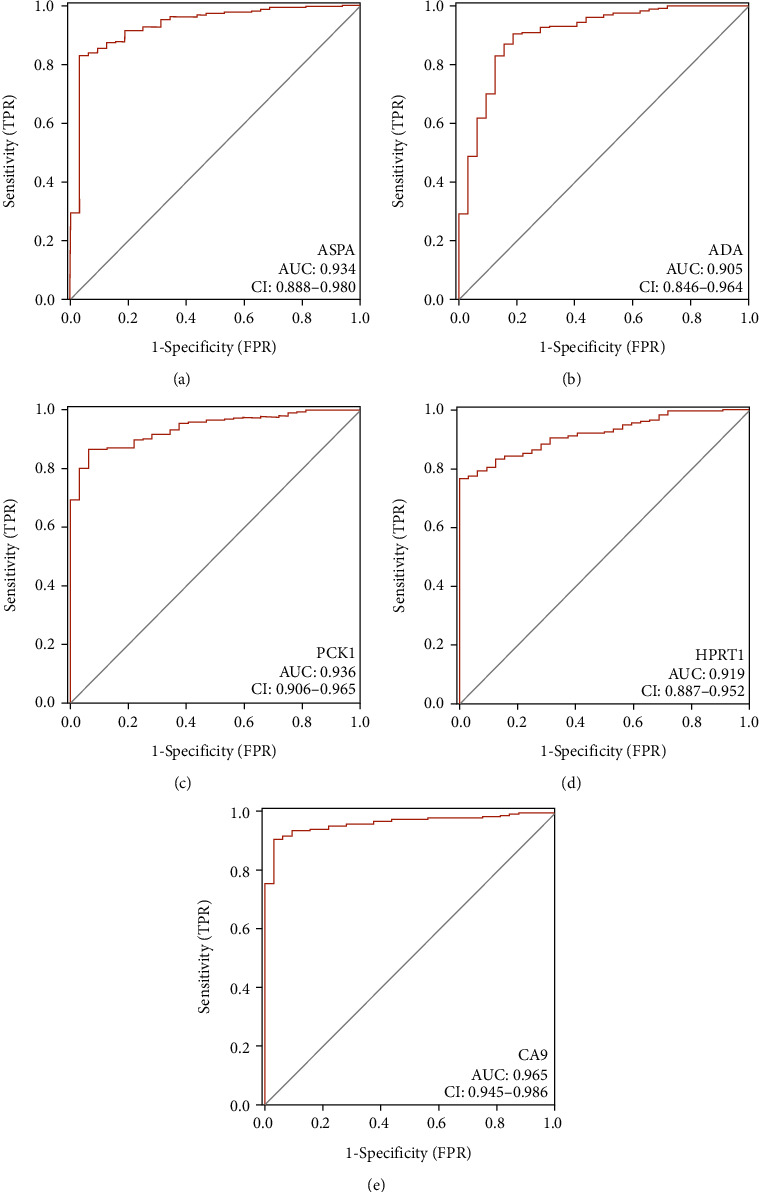
Identification of key genes for the diagnosis of OSCC. Genes with an AUC value of >0.85 are shown in (a)–(e).

ROC curve analysis revealed that among the 8 genes in the prognostic signature, ADA, HPRT1, CA9, ASDA, and PCK1 achieved an AUC value of >0.85 and had high sensitivity and specificity for OSCC diagnosis (Figure 4); these results indicated that ADA, HPRT1, CA9, ASDA, and PCK1 had potential diagnostic value in OSCC.

### 3.5. Selection of Prognostic Biomarkers of OSCC

**Figure 5:**
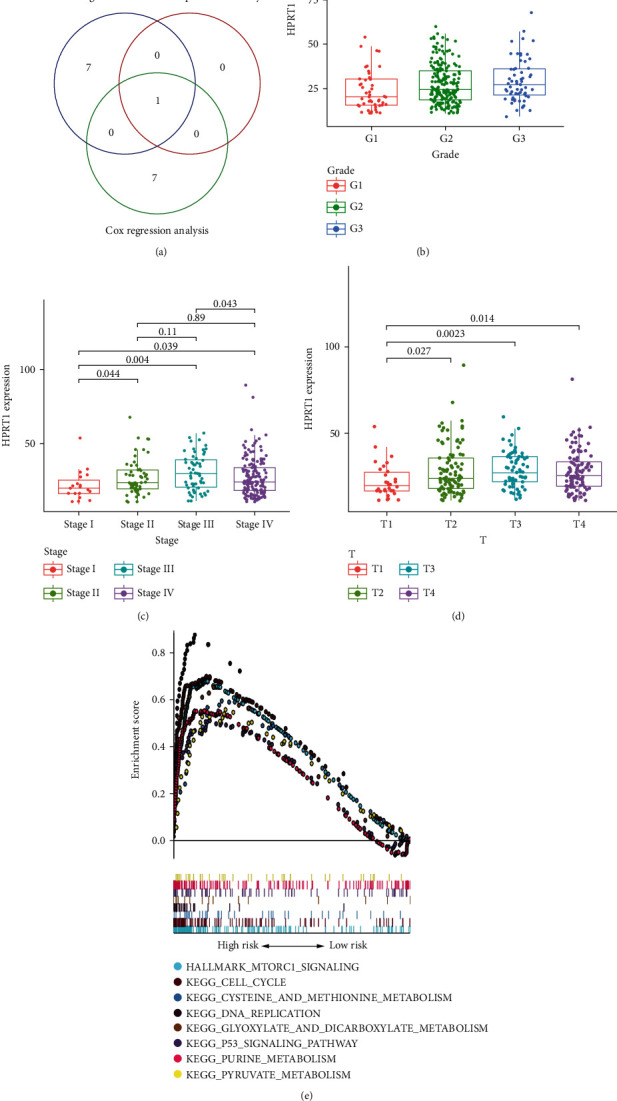
Selection of prognostic biomarkers. (a) Venn diagram to search biomarkers. (b) Relationship between HPRT1 expression and grade. (c) Relationship between HPRT1 expression and stage. (d) Relationship between HPRT1 expression and T classification. (e) Enrichment analysis of HPRT1 by GSEA.

The intersection of Cox regression analysis (Supplement Figures 1A and 1D), Kaplan-Meier analysis (Supplement Figure 3), and hub genes (Figure 1(e)) revealed that only HPRT1 was a potential biomarker of OSCC (Figure 5(a)). Further analysis found that HPRT1 mRNA expression was significantly related to pathological grade (Figure 5(b)), clinical stage (Figure 5(c)), and T classification (Figure 5(d)). However, the expression of HPRT1 decreased in patients with stage IV and T4 disease, which requires further study. Using GSEA, we discovered functions and biological pathways associated with low and high HPRT1 expression levels in TCGA expression datasets. The pathways were considered significant with the standard of |*NES*| > 1, NOM < 0.05, and FDR < 0.05. The results showed that the high HPRT1 expression phenotype was associated with numerous vital pathways related to tumorigenesis, including the mTORC1 signaling pathway, P53 signaling pathway, cell cycle pathway, DNA replication pathway, glyoxylate and dicarboxylate metabolism pathway, purine metabolism pathway, and pyruvate metabolism pathway (Figure 5(e)).

### 3.6. Exploration of the Biological Functions of HPRT1 In Vitro

**Figure 6:**
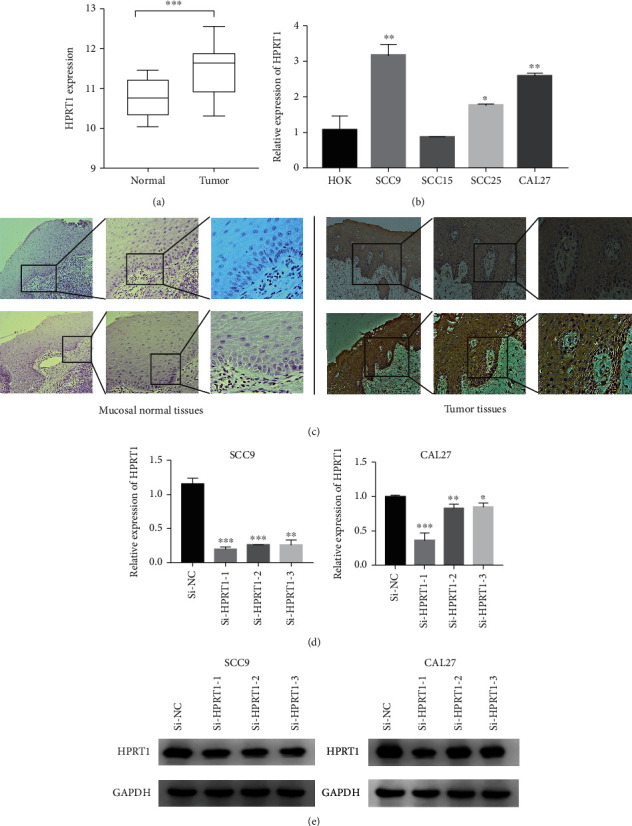
Validation of HPRT1 expression. (a, b) Validation of HPRT1 mRNA expression by real-time PCR in 20 pairs of tissues and normal tissues as well as 4 OSCC cell lines. Human oral keratinocyte cells (HOKs) were used as a control. (c) The representative results of immunohistochemistry. (d) Confirmation of HPRT1 mRNA expression levels in SCC9 and CAL27 cells after transfection. (e) The protein expression of HPRT1 in SCC9 and CAL27 cells was measured by Western blotting. ^∗^*p* < 0.05, ^∗∗^*p* < 0.01, and ^∗∗∗^*p* < 0.001.

**Figure 7:**
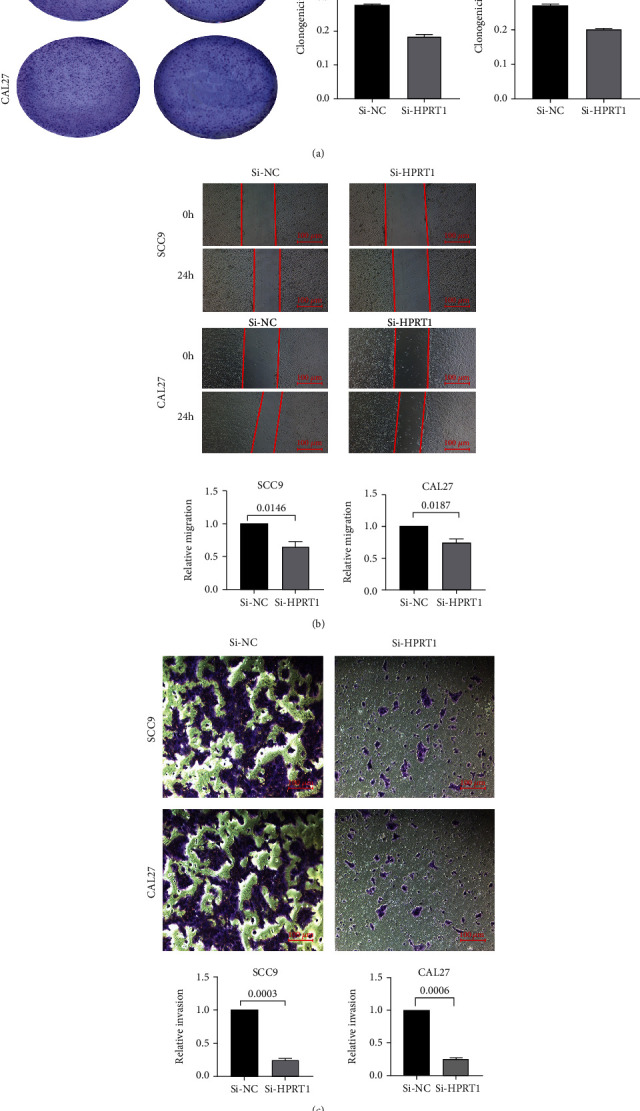
Functional experiment in vitro. (a) The proliferation of SCC9 and CAL27 cells was measured by a colony formation assay. (b) Inhibiting HPRT1 expression inhibited the migration of OSCC cells. (c) Inhibiting HPRT1 expression inhibited the invasion of OSCC cells.

The mRNA expression levels of HPRT1 were evaluated in 20 pairs of OSCC tissues and normal tissues as well as in four oral squamous cell cancer cell lines. The results suggested that HPRT1 mRNA expression was higher in OSCC tissues than in normal tissues and higher in the OSCC cell lines than in the HOK cell line (Figures 6(a) and 6(b)). Then, the protein expression of HPRT1 in OSCC tumors was found to be higher than that in paired normal tissues by the immunohistochemistry assay (Figure 6(c)). HPRT1 is mainly distributed in the cytoplasm. Since higher HPRT1 expression levels were positively correlated with the advanced clinical stage (Figure 3(c)), we decided to investigate whether HPRT1 affected the proliferation, invasion, and migration of OSCC cells. The SCC9 and CAL27 cell lines were selected for this experiment. HPRT1 expression was silenced with siRNA in SCC9 and CAL27 cells. The transfection efficiency of siRNA against HPRT1 was measured by real-time PCR and Western blotting assays (Figures 6(d) and 6(e)). The results showed that silencing HPRT1 expression suppressed the proliferation, migration, and invasion of SCC9 and CAL27 cells, as shown by colony formation, wound healing, and Transwell assays (Figures 7(a)–7(c)).

## 4. Discussion

OSCC, the most common type of oral cancer, presents a significant challenge to the medical community due to its high recurrence rate and low 5-year overall survival rate [22]. In recent years, there has been a marked increase in interest in the dysregulation of metabolism in cancer [23]. According to accumulating data [24], MRGs have been shown to play a critical role in the development and progression of cancer. Therefore, it is necessary to explore the critical MRGs that could be used as prognostic biomarkers and therapeutic targets for OSCC [25].

In this study, a total of 170 DEMRGs (differentially expressed MRGs) were identified in TCGA dataset; of these DEMRGs, 104 MRGs were downregulated and 66 MRGs were upregulated. According to GO analysis, the DEMRGs were enriched in biological processes such as iron ion binding, oxidoreductase activity, and oxygen coenzyme binding, which play crucial roles in tumorigenesis. In addition, KEGG pathway analysis showed that DEMRGs were mainly enriched in tyrosine metabolism, arginine and proline metabolism, and fatty acid degradation pathways. Among these pathways, the tyrosine metabolism pathway and the arginine and proline metabolism pathways are significantly related to different types of cancer. The phosphorylation of tyrosine is a ubiquitous posttranslational modification that is important for the metabolic reprogramming of cancer cells [26]. In addition, studies have revealed that the arginine and proline metabolism pathways are relevant to the proliferation and metastasis of human prostate cancer [27].

Univariate Cox regression analysis revealed that 9 MRGs were related to overall survival in OSCC, which indicated that MRGs might play an important role in OSCC carcinogenesis. An innovative prognostic signature consisting of 8 MRGs (ASPA, HPRT1, CA9, ADH7, AGPAT4, CHDH, ADA, and PCK1) was established by LASSO regression and multivariable Cox regression analysis. OSCC patients were divided into high-risk and low-risk groups based on their gene signatures. Patients in the high-risk group had significantly shorter overall survival than those in the low-risk group. According to time-dependent ROC analysis, it was determined that the AUCs for 1-, 2-, and 3-year OS were 0.761, 0.727, and 0.744, respectively. These results suggested that the prognostic signature has some value in predicting tumor prognosis. This signature's prognostic value was successfully verified in the validation set. Further study showed that this signature might be an independent prognostic predictor.

ROC analysis revealed that 5 of these genes (ADA, PCK1, HPRT1, CA9, and ASPA) were potential molecular markers for the diagnosis of OSCC due to their relatively high sensitivity and specificity. PCK1, CA9, ADA, and ASPA were found to exert carcinogenic effects in a metabolism-dependent manner in various malignancies. PCK1 is the rate-limiting enzyme of gluconeogenesis, and its expression is frequently increased in individuals with metabolic syndrome and diabetes mellitus [28]. Liu et al. found that PCK1 gene expression is downregulated in primary hepatocellular carcinoma (HCC) and that low PCK1 expression is associated with a poor prognosis in HCC patients [29]. In oral cancer, PCK1 expression is also downregulated. Carbonic anhydrase 9 (CA9) belongs to the carbonic anhydrase family and is a transmembrane enzyme. CA9 is overexpressed in urinary bladder cancer and is a potentially promising diagnostic marker for the disease [30]. We discovered that CA9 expression was similarly elevated in OSCC. ADA was also reported to play an oncogenic role in hepatocellular carcinoma [31]. Regrettably, few studies have indicated that these MRGs are associated with OSCC carcinogenesis. Further research is needed to determine the roles of these genes in OSCC.

By Kaplan-Meier analysis, we found that only HPRT1 has the potential to predict the prognosis of OSCC patients. HPRT1 is essential in providing building blocks for future cell growth. It is a housekeeping gene widely used as an endogenous control in gene expression studies [32]. However, recent research revealed that it might play a role in carcinogenesis. The expression level of HPRT1 increased across many cancer types [33]. HPRT1 expression was found to be upregulated in head and neck squamous cell carcinoma and might be a promising prognostic indicator and treatment target for HNSCC [34]. Nevertheless, the biological functions of HPRT1 in OSCC remain unclear. Our results confirmed that HPRT1 expression was upregulated in OSCC tissues and positively correlated with advanced clinical stage. Furthermore, our research indicated that HPRT1 promoted the proliferation, invasion, and migration of OSCC in vitro, which revealed that HPRT1 might play an essential role in OSCC carcinogenesis. GSEA showed that HPRT1 was significantly enriched in pathways and essential biological functions related to tumorigeneses, such as the mTORC1 signaling pathway, P53 signaling pathway, and cell cycle pathway.

In the current research, we developed a risk model based on the expression of MRGs. Additionally, the risk score may be an independent predictive biomarker according to univariate and multivariate Cox regression analyses. Finally, survival analysis showed that HPRT1 might contribute to the tumorigenesis of OSCC. By confirming the expression levels and functions of HPRT1 in OSCC cells, it was determined that HPRT1 enhanced cell proliferation, migration, and invasion in vitro.

However, we must acknowledge that the current study has limitations that should be addressed in future research. First, because the transcriptome and clinical data of patients with OSCC were collected from a public database, potential selection bias could not be ruled out. Second, the function of these biomarkers in OSCC, as well as the underlying mechanisms, needs to be further studied in vivo.

## Data Availability

The data that support the findings of this study are available in TCGA database. These data were derived from the following resources available in the public domain: https://portal.gdc.cancer.gov/.

## Supplementary Materials

Supplementary Materials Supplement Figure 1: (A) Univariate cox regression analysis on MRGs. (B, C) Lasso coefficient profiles of the 9 MRGs with nonzero coefficients determined by the optimal lambda. (D) Multivariate cox analysis to developing a prognostic signature based on these MRGs. Supplement Figure 2: mRNA levels in paired adjacent normal tissues and OSCC tissues from GSE37991. The results showed that each gene was differentially expressed, which was consistent with the results in TCGA database. ^∗^*p* < 0.05, ^∗∗^*p* < 0.01, and ^∗∗∗^*p* < 0.001. Supplement Figure 3: Kaplan-Meier analysis and a log-rank test to examine the OS of OSCC patients in TCGA data set.
