# Supplementary material for: Metabolism-Related Bioinformatics Analysis Reveals That HPRT1 Facilitates the Progression of Oral Squamous Cell Carcinoma In Vitro
Source: J Oncol. 2022 May 9;2022:7453185. doi: 10.1155/2022/7453185 (PMC9110220; doi:10.1155/2022/7453185)
Supplement: Supplementary Materials — Supplement Figure 1: (A) Univariate cox regression analysis on MRGs. (B, C) Lasso coefficient profiles of the 9 MRGs with nonzero coefficients determined by the optimal lambda. (D) Multivariate cox analysis to developing a prognostic signature based on these MRGs. Supplement Figure 2: mRNA levels in paired adjacent normal tissues and OSCC tissues from GSE37991. The results showed that each gene was differentially expressed, which was consistent with the results in TCGA database. ∗p < 0.05, ∗∗p < 0.01, and ∗∗∗p < 0.001. Supplement Figure 3: Kaplan-Meier analysis and a log-rank test to examine the OS of OSCC patients in TCGA data set. [file 7453185.f1.docx]

**Supplement Figures**


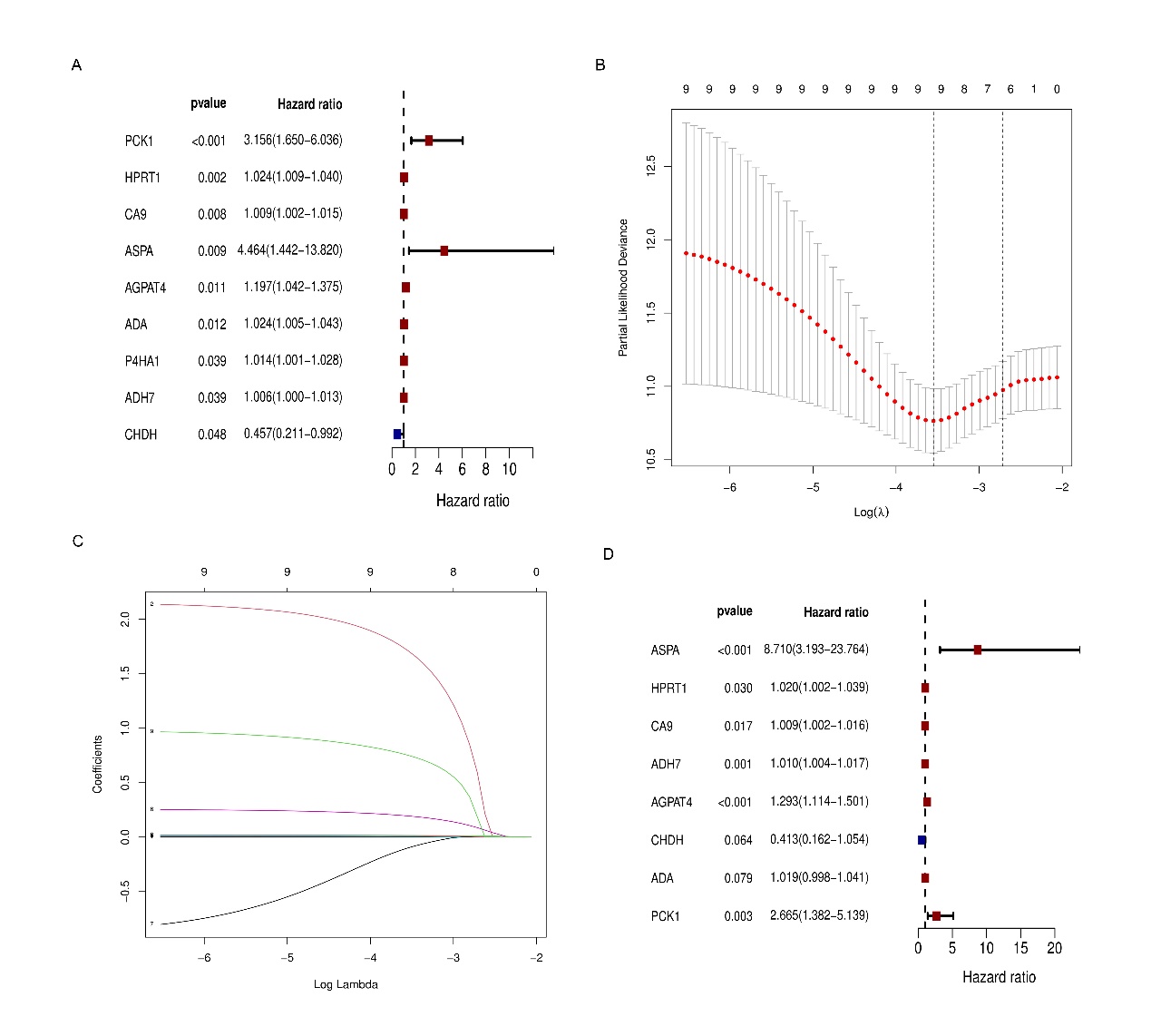


**Supplement Fig.1 (A)** Univariate cox regression analysis on MRGs. **(B)**&**(C)** Lasso coeficient profiles of the 9 MRGs with non-zero coefficients determined by the optimal lambda. **(D)** Multivariate cox analysis to developing a prognostic signature based on these MRGs.


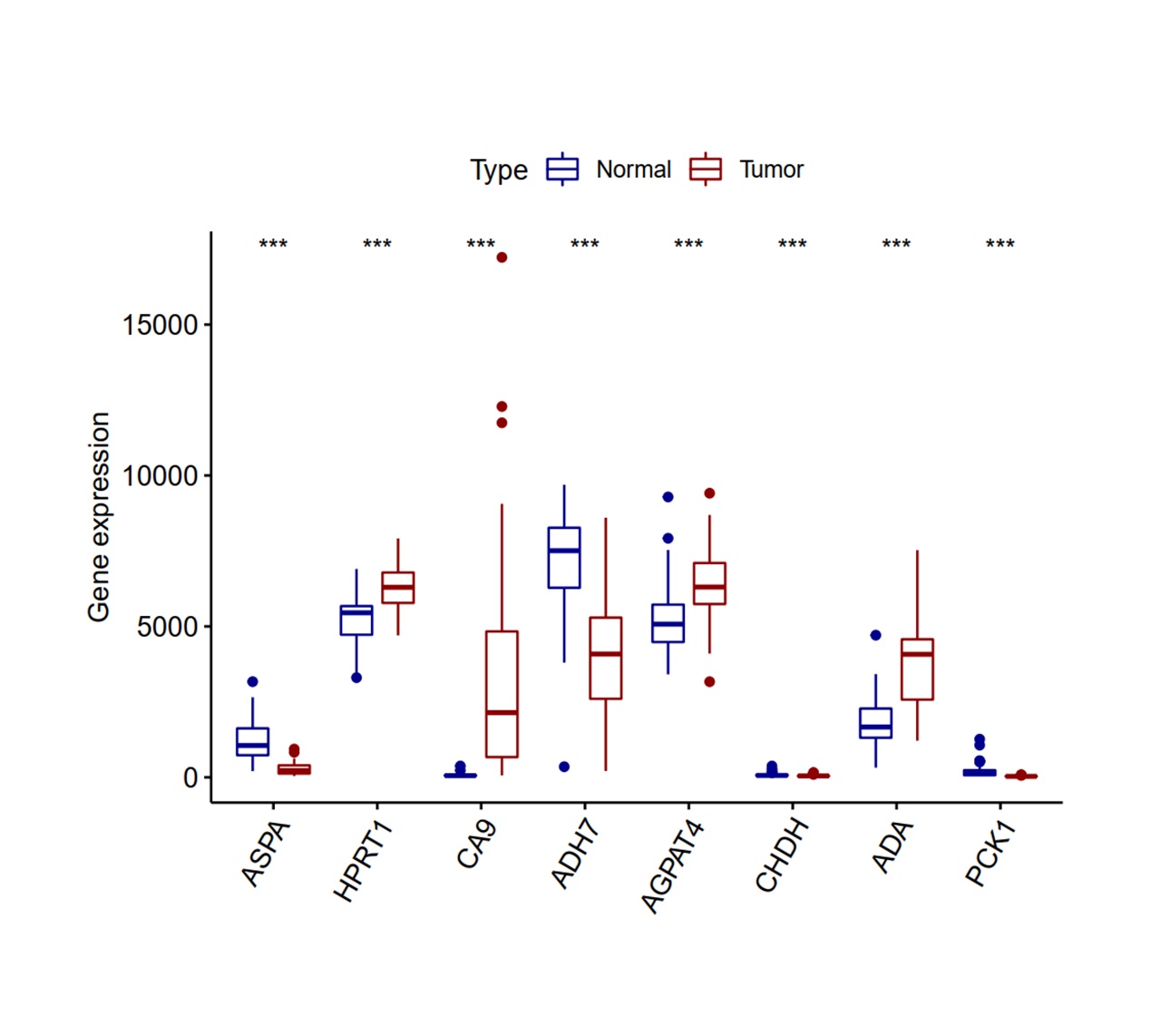


**Supplement Fig.2** mRNA levels in paired adjacent normal tissues and OSCC tissues from GSE37991. The results showed that each gene was differentially expressed, which was consistent with the results in TCGA database. * p < 0.05, ** p < 0.01, *** p < 0.001.


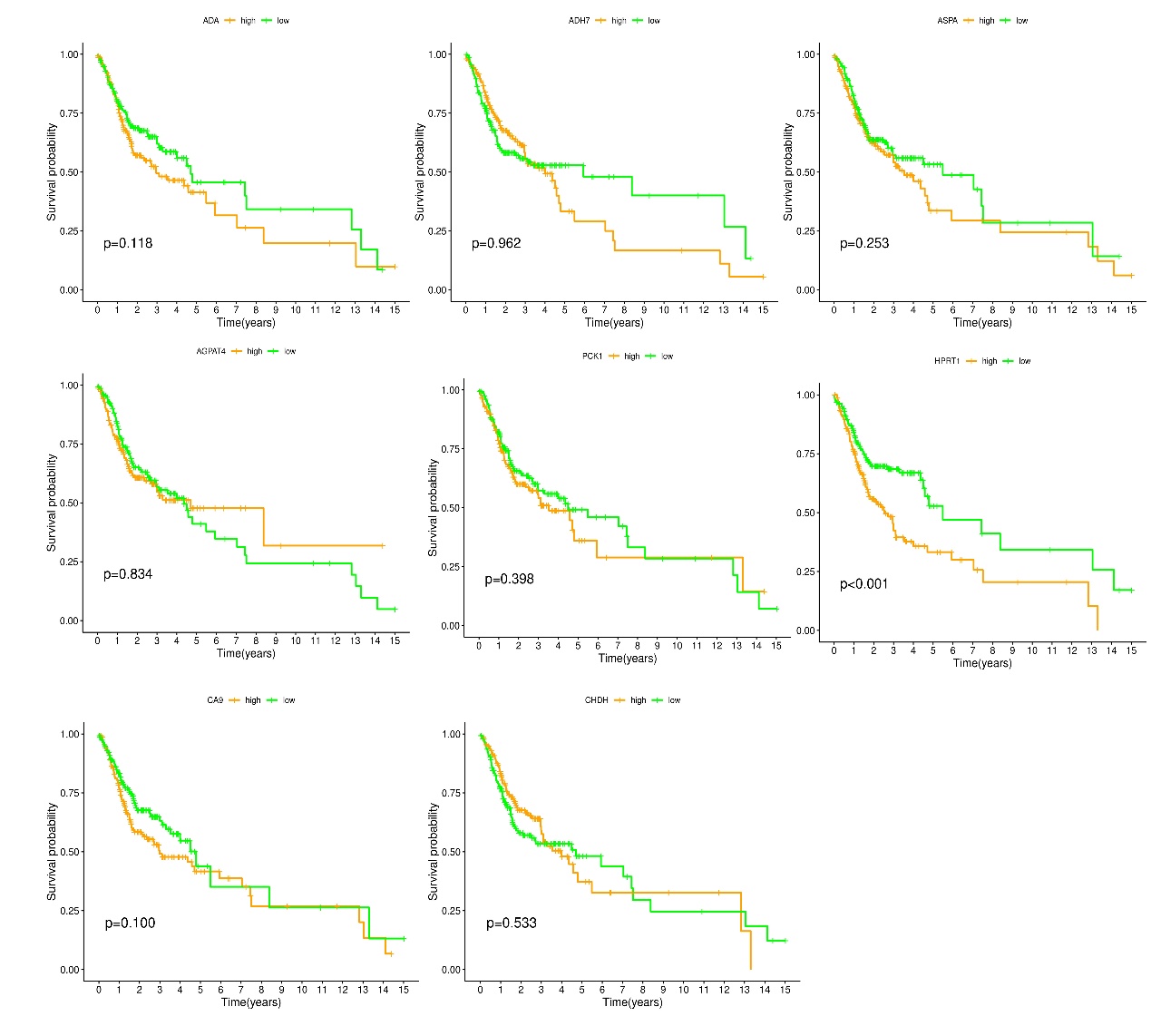


**Supplement Fig.3** Kaplan-Meier analysis and a log-rank test to examine the OS of OSCC patients in TCGA data set.
